# *Phasertng*: directed acyclic graphs for crystallographic phasing

**DOI:** 10.1107/S2059798320014746

**Published:** 2021-01-01

**Authors:** Airlie J. McCoy, Duncan H. Stockwell, Massimo D. Sammito, Robert D. Oeffner, Kaushik S. Hatti, Tristan I. Croll, Randy J. Read

**Affiliations:** a Department of Haematology, Cambridge Institute for Medical Research, University of Cambridge, Hills Road, Cambridge CB2 0XY, United Kingdom; b Drug Discovery Unit, Wellcome Centre for Anti-Infectives Research, School of Life Sciences, University of Dundee, Dow Street, Dundee DD1 5EH, United Kingdom

**Keywords:** *Phaser*, *Phasertng*, molecular replacement, SAD phasing, directed acyclic graphs, maximum likelihood, machine learning

## Abstract

Crystallographic phasing strategies increasingly require the exploration and ranking of many hypotheses about the number, types and positions of atoms, molecules and/or molecular fragments in the unit cell, each with only a small chance of being correct. Accelerating this move has been improvements in phasing methods, which are now able to extract phase information from the placement of very small fragments of structure, from weak experimental phasing signal or from combinations of molecular replacement and experimental phasing information. Describing phasing in terms of a directed acyclic graph allows graph-management software to track and manage the path to structure solution. The crystallographic software supporting the graph data structure must be strictly modular so that nodes in the graph are efficiently generated by the encapsulated functionality. To this end, the development of new software, *Phasertng*, which uses directed acyclic graphs natively for input/output, has been initiated. In *Phasertng*, the codebase of *Phaser* has been rebuilt, with an emphasis on modularity, on scripting, on speed and on continuing algorithm development. As a first application of *phasertng*, its advantages are demonstrated in the context of *phasertng.xtricorder*, a tool to analyse and triage merged data in preparation for molecular replacement or experimental phasing. The description of the phasing strategy with directed acyclic graphs is a generalization that extends beyond the functionality of *Phasertng*, as it can incorporate results from bioinformatics and other crystallographic tools, and will facilitate multifaceted search strategies, dynamic ranking of alternative search pathways and the exploitation of machine learning to further improve phasing strategies.

## Abstract 2

The employment of directed acyclic graphs to advance the tracking, control and appraisal of crystallographic phasing strategies is discussed.

## 1. Introduction   

Our *Phaser* crystallographic software for phasing macromolecular crystal structures based on maximum likelihood and multivariate statistics (Bricogne, 1992 ▸, 1997 ▸; Read, 2001 ▸) has been an asset to the crystallographic community, having solved tens of thousands of macromolecular crystal structures in the Protein Data Bank (PDB; Burley *et al.*, 2019 ▸). The focus of our developments has been phasing by molecular replacement (MR; Huber, 1965 ▸; Read, 2001 ▸) and single-wavelength anomalous dispersion (SAD; Hendrickson & Teeter, 1981 ▸; Pannu & Read, 2004 ▸) because these methods are similar in having the relative ease of requiring only a single (merged) data set, because they are both amenable to rigorous likelihood treatments and because single-wavelength data collection can require a lower total radiation dose than multiple-wavelength methods. Most of the structures deposited in the PDB are currently phased by one or the other of these two methods (Burley *et al.*, 2019 ▸). However, both MR and SAD phasing can fail for unavoidable reasons, and phasing by multiple isomorphous replacement (MIR; Green *et al.*, 1954 ▸; Blow & Crick, 1959 ▸), multiple-wavelength anomalous dispersion (MAD; Hendrickson, 2014 ▸; Hendrickson & Teeter, 1981 ▸) or multiple isomorphous replacement with anomalous scattering (MIRAS; Vonrhein *et al.*, 2007 ▸; Rossmann, 1961 ▸) are alternatives to MR and SAD that should be included in broader phasing strategies.

Highlights of the ongoing development in *Phaser* have been the fast maximum-likelihood rotation function, the fast maximum-likelihood translation function, SCEDS domain analysis through normal-mode perturbation, ensemble variance estimation and refinement, translational noncrystallographic symmetry (TNCS) expected intensity-correction terms, twinning detection in the presence of translational non­crystallographic symmetry, the log-likelihood gain on intensity, single-atom MR, *gyre* and *gimble* refinement, *Phassade* substructure determination, information content and translational noncrystallographic symmetry detection [for a review, see McCoy (2017 ▸) and citations therein].

In addition, the introduction of the expected log-likelihood gain (eLLG; McCoy *et al.*, 2017 ▸) in *Phaser* has brought about a fundamental change in MR strategies. With the eLLG, it has become possible to calculate the probability that MR with a given model will succeed, replacing the *ad hoc* rules that have guided the attempts of crystallographers to predict the outcome of MR, and to prove that rules based solely on minimum percentages of sequence identity between the model and the target are not sufficient for good prediction across all resolution ranges and model sizes. Using the eLLG as a guide, minimal models can be prepared with the confidence that a solution is possible within the resources available. For phasing problems that are amenable to this approach, successful MR can be achieved with models consisting of small units of secondary structure (Glykos & Kokkinidis, 2003 ▸; Robertson *et al.*, 2010 ▸), conserved cores of structurally divergent proteins (Bernstein *et al.*, 1997 ▸) or reliable fragments of *ab initio* models (Qian *et al.*, 2007 ▸). Fragment-based approaches to model generation and MR have proven to be highly effective (Rodríguez *et al.*, 2009 ▸; Bibby *et al.*, 2012 ▸) and the use of small fragments for MR, with many such fragments needing to be placed, is now well established.

MR has also benefitted from advances in homology modelling and *ab initio* modelling (Kryshtafovych *et al.*, 2019 ▸). Utility for MR was a scoring criterion in CASP13 (Read *et al.*, 2019 ▸; Croll *et al.*, 2019 ▸), with contributors being encouraged to deposit not only coordinates but also estimates of coordinate error. Accurate coordinate-error estimates have been demonstrated to improve success in MR calculations (Bunkóczi *et al.*, 2015 ▸). When only very poor templates are available, CASP13 showed that the best homology models are better than the best template or even the best ensemble from PDB entries (Wallner, 2020 ▸; Croll *et al.*, 2019 ▸). Contributing to these improvements has been the incorporation of evolutionary-covariance information in the modelling process (Simkovic *et al.*, 2016 ▸). The key to these implementations of MR strategy is the generation of many models, each slightly perturbed from the others, so that as a group they sample conformational space finely enough that at least one is able to model the target sufficiently for a MR signal to be obtained. With the multi-trial approach to MR, data tracking becomes a significant part of the phasing strategy.

These approaches of extracting solutions from many phasing attempts, each individually with a low probability of success, but with a high probability of overall success, have also partly been driven by Moore’s law rates of increase in processing speed and the increasing number of CPUs available on the desktop (Waldrop, 2016 ▸).

We have come to realize that further development of our phasing strategies will require a step change in the software from our laboratory. We describe here how the source code of *Phaser* has been rebuilt as *Phasertng* in order to make use of advances in computing and to meet user expectations of faster and more automated software that can optimally explore a wide range of structure-solution strategies.

## 2. Directed acyclic graphs   

**Figure 1:**
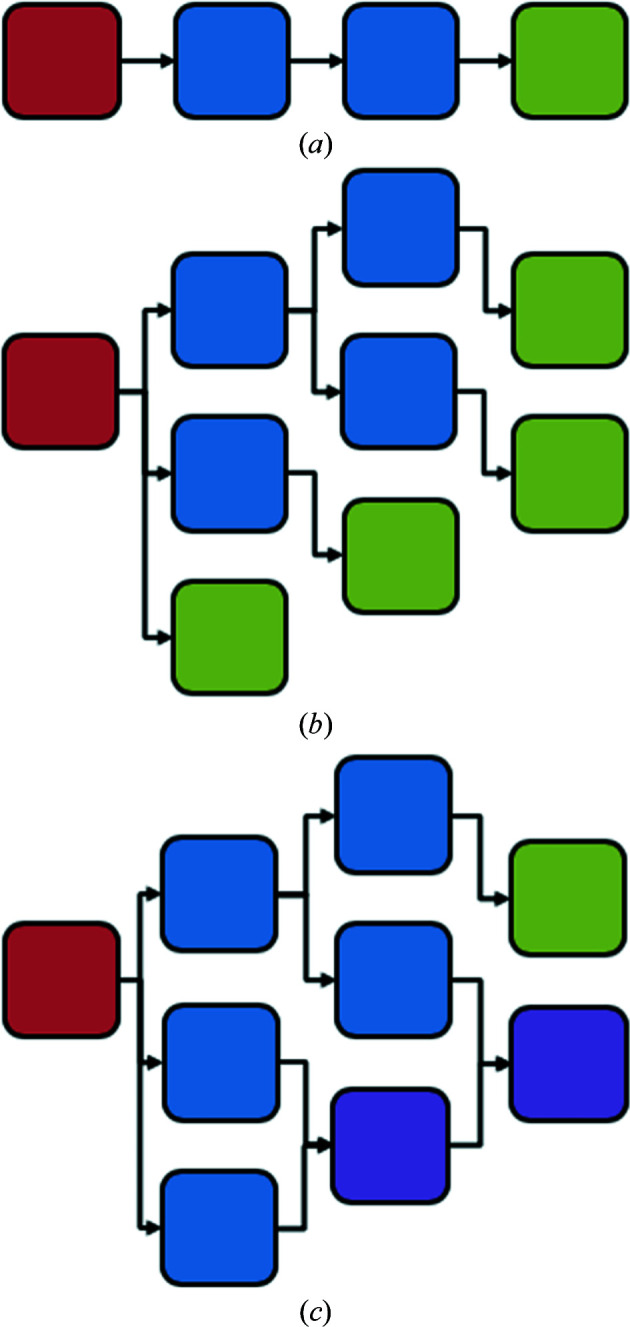
(*a*) Path, (*b*) tree, (*c*) directed acyclic graph. The root node is shown in red, leaf nodes are shown in green and intermediate are shown in blue, except that all nodes with two parents are shown in purple.

A directed acyclic graph (DAG) is a type of graph that describes an aetiological network linking causes to effects. Formally, a DAG (Fig. 1 ▸) is a finite graph in which the edges are directed and there are no directed cycles, and nodes can have more than one parent. DAGs underly dataflow programming, where ‘the ordering of the operations is not specified by the programmer, but … is implied by the data interdependencies’ (Sharp, 1992 ▸). The DAG describes the connections between operations rather than the order in which they should occur; upon the execution of a dataflow program, the computer infers the order of operation from the connections given in the DAG. An early application of DAGs in computing was the visual programming language Prograph (Matwin & Pietrzykowski, 1985 ▸) written for the Apple Macintosh.

Our choice of directed acyclic graphs for describing phasing pathways is supported by our experience of automation in *Phaser*. The tree-search-with-pruning strategy for MR and SAD in *Phaser* (McCoy *et al.*, 2007 ▸) makes effective use of the strength of the maximum-likelihood functions in using prior information in the search for additional components in the asymmetric unit: either MR models or anomalously scattering atoms. The tree-search-with-pruning strategy is formally a directed acyclic graph.

We define the nodes of the DAG as hypotheses for the unit cell, with the edge direction describing increasing information about the position of atoms within. The data encompass information about the crystallization-drop contents, data processing, crystal symmetry, SAD substructures, partial or full poses of models during MR and validated atomic models of the asymmetric unit. The data structure of the node is extensible. Nodes also contain information about the reliability or ranking of the hypothesis.

Nodes with more than one parent can arise in several different scenarios in phasing. Perhaps the most significant is at the stage of placing components by MR, where several poses of (the same or different) components, identified by independent rotation and translation functions and therefore independent hypotheses for the contents of the asymmetric unit, may be brought together (on the same origin) to build a combined hypothesis for the contents of the asymmetric unit. Other examples of scenarios where nodes are combined from two parents include the validation of MR model placements with independently determined SAD substructures, or where placed MR components are substituted with homologous components and rescored to find the best components for phasing.

## 3. Development of *Phasertng*   

The core functionality in *Phaser* has been reconfigured as *Phasertng*, principally to support the DAG framework, but the opportunity to rebuild the codebase has allowed us to make other improvements. The development of new algorithms has continued throughout this process.

*Phasertng* retains the popular features of *Phaser*, including the ability to run either as binary executables or as Python modules. Almost unchanged from *Phaser* is the method of logging text output, including logfile output of different levels of verbosity, keywords that toggle the writing of files and callbacks to Python to provide updates on progress.

*Phasertng* differs from *Phaser* in four major ways: a modular architecture to encapsulate the functionality that generates DAG nodes; the use of Phil files for input and output (Echols *et al.*, 2012 ▸) to support scripting; the use of enhanced features of C++11 over C++98, including the use of the C++11 standard threading library (ISO, 1998 ▸, 2011 ▸) to increase speed; and last, but certainly not least, improved algorithms. We expand on these four ways below.

### 3.1. DAG modularity   

We retain the term ‘mode’ previously used to refer to *Phaser*’s different executable blocks, but whereas in *Phaser* a ‘mode’ does not cleanly represent a single functionality, in *Phasertng* the software is strictly modular. Each ‘mode’ generates a branch on the DAG, and can be initiated with the information contained in, and only in, the DAG nodes. The strict modularity means that data-preparation steps do not need to be repeated in subsequent steps, and the restarting of structure solution from a halted pathway is trivial and transparent.

### 3.2. Scripting   

*Phasertng* has input and output in the Python-based hierarchical interchange language Phil (Echols *et al.*, 2012 ▸). By using Phil for output, results are available in Python. By also using Phil files as input, parameters set by one mode can be used by another without the need for reformatting. Keyword documentation (including information about the defaults) is generated from a master Phil file, which ensures that the code and documentation are synchronized. Coordinate data input/output in *Phasertng* uses PDB-format files, and reflection data are input/output using MTZ-format files (Winn *et al.*, 2011 ▸).

### 3.3. Speed   

The *Phasertng* code has been parallelized using the ‘thread’ and ‘future’ libraries introduced with the C++11 ISO standard (ISO, 2011 ▸). The threading is implemented where a computationally expensive function evaluation must be calculated for each reflection; the threading is over the reflection loop. Although parts of the *Phaser* code were parallelized with the nonstandard OpenMP library (Dagum & Menon, 1998 ▸), the granularity of the parallelization was coarser than that in *Phasertng*, owing to the overhead in initializing OpenMP threads, and the threading was not implemented over reflection loops. Profiling of the source code with *Gprof* (Graham *et al.*, 2004 ▸) has led to further increases in speed.

### 3.4. Improved algorithms   

Mathematical derivations of the functions included in *Phasertng* were published approximately contemporaneously with their release in *Phaser*; however, inspection of the source code in *Phaser* and *Phasertng* will show variation from the functions as published. Contributing to the variation has been the adoption of the log-likelihood gain on intensity (LLGI; Read & McCoy, 2016 ▸) and the addition of TNCS-correction terms (Sliwiak *et al.*, 2014 ▸) throughout the source code. Most of the functions are not only used for function evaluation but are also used as refinement targets. In implementing functions for refinement, the parameterization of the functions is important and the parameterizations have been stringently tested for robust convergence and numerical stability. The minimization code itself has also been developed to handle the specific features of the likelihood functions, which include correlated parameters and parameters on very different scales (Stockwell *et al.*, 2020 ▸). Thus, the public release of the source code is the most complete, up-to-date and exhaustive form of publication of our methods.

## 4. *phasertng.xtricorder*   

As an example of the functionality of *Phasertng*, we describe the implementation of *phasertng.xtricorder*, which is a data-analysis and preparation tool that provides some functionality overlapping with *phenix.xtriage* (Zwart *et al.*, 2005 ▸) and *TRUNCATE* in *CCP*4 (Winn *et al.*, 2011 ▸). In the context of our phasing strategies in development, *phasertng.xtricorder* is needed in order to provide appropriate data preparation so as to optimize the capabilities of our maximum-likelihood phasing; the underlying maximum-likelihood functions are highly dependent on appropriate multi-step preparation of the data for optimal performance. Since the introduction of the LLGI target (Read & McCoy, 2016 ▸), data for maximum-likelihood calculations should preferentially be entered as intensities and should not have been through the French and Wilson procedure (French & Wilson, 1978 ▸) that yields posterior expectations. Externally applied anisotropic data truncation is extremely problematic for our algorithms, because the truncation hampers correct normalization of the data. Where the LLGI target is employed, low information-content reflections can be excluded internally, on-the-fly, in the interest of speed (Jamshidiha *et al.*, 2019 ▸).

The *phasertng.xtricorder* tool reads the data from an input merged MTZ-format data file; analyses the data to determine whether the French and Wilson procedure (French & Wilson, 1978 ▸) has been applied to the data and generates reflection intensities; performs anisotropy correction; finds the possible TNCS order(s); performs TNCS correction for the TNCS order(s); estimates the probability that the data are twinned; and expands the data to subgroups if twinning is detected. In the general case, the result of running *phasertng.xtricorder* is a set of MTZ-format data files with different TNCS orders, TNCS corrections and space-group expansions. In the simplest case, where TNCS and twinning are absent, there will only be a single MTZ-format data file. These data files are ready for taking forward into MR and SAD phasing trials with maximum-likelihood functions.

The *phasertng.xtricorder* tool is most closely related to *Phaser*’s NCS mode. The *phasertng.xtricorder* tool expands on the functionality in *Phaser*’s NCS mode by carrying out the Padilla–Yeates *L*-test (Padilla & Yeates, 2003 ▸) using the TNCS intensity-correction terms (Sliwiak *et al.*, 2014 ▸) and performing space-group expansion. In addition, *phasertng.xtricorder* has modifications to details of the anisotropy correction [the replacement of Newton’s method of refinement with BFGS refinement (Fletcher, 1987 ▸) and changes to the restraint terms at low resolution], changes to the parameterization of the TNCS correction (effective molecular radius of volume related by TNCS, r.m.s. deviation between TNCS-related components and resolution-dependent fraction of the scattering related by TNCS) and modifications to the minimiser code (Stockwell *et al.*, 2020 ▸). In *phasertng.xtricorder*, the reflection loops for the calculation of the anisotropy-correction terms, the TNCS correction terms and the outlier rejection have been parallelized with the C++11 threading library.

We illustrate the advantages of *Phasertng* over *Phaser* in two different ways: firstly by showing the tracking capabilities of the DAG infrastructure and secondly by providing speed comparisons.

### 4.1. DAG   

The results of the *phasertng.xtricorder* tool are recorded as nodes in a DAG data structure. There are no cycles and so the data structure is a ‘tree’ subgroup of the DAG (Fig. 1 ▸). By comparison, *Phaser*’s NCS mode uses only the most probable TNCS order for TNCS correction and does not expand to subgroups if twinning is detected, and so the results can be described as a ‘path’ subgroup of the DAG (Fig. 1 ▸), although the results are not reported in this form.

**Figure 2:**
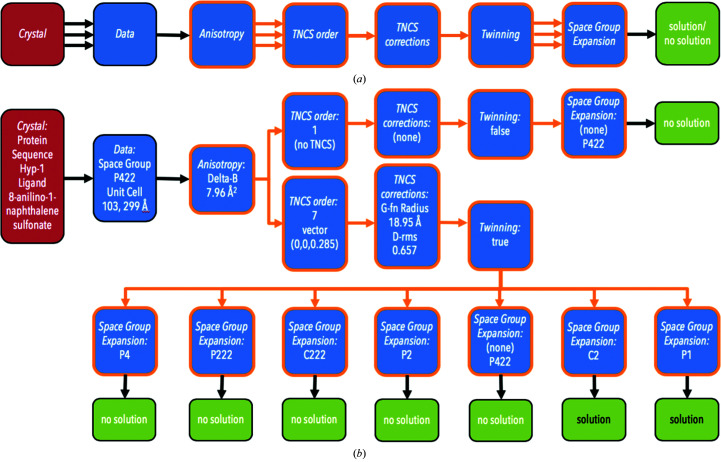
(*a*) Schematic for the DAG resulting from user input and *phasertng.xtricorder* with a colouring scheme as in Fig. 1 ▸. Nodes outlined in orange are those generated by *phasertng.xtricorder*. Multiple arrows indicate where the DAG may branch. (*b*) Schematic of DAG for structure solution of PDB entry 4n3e (Sliwiak *et al.*, 2014 ▸). The crystal was obtained by co-crystallization of Hyp-1 with an eightfold molar excess of the ligand 8-anilino-1-naphthalene sulfonate (ANS). Strong fluorescence under UV illumination confirmed the presence of ANS in the crystals. Data were collected from a single crystal at a wavelength of 1.00 Å. The data extended to 2.4 Å resolution. There were no systematic absences of reflections along the axes and the highest symmetry in which the data merged was space group *P*422. Significant anisotropy was present. TNCS of order 7 was suspected as a result of analysis of the Patterson map, with the absence of TNCS maintained as a hypothesis. Twinning was detected in the intensity statistics after TNCS corrections for order 7. Since overmerging is possible in the presence of twinning, data were expanded to all subgroups of *P*422. Twinning was not detected in the absence of TNCS. At the conclusion of *phasertng.xtricorder* data analysis there are eight hypotheses for the contents of the asymmetric unit; solutions can be obtained for two of these hypotheses.

Fig. 2 ▸ shows an example of the DAG nodes generated by *phasertng.xtricorder* for PDB entry 4n3e (Sliwiak *et al.*, 2014 ▸), which is a case of a highly pathological crystal with tetartohedral twinning and sevenfold TNCS. This structure was solved by taking the data merged in *P*422, and after suspecting twinning, expanding the data to *P*1 for MR. MR was performed with TNCS of order 7 and finding 56 monomers in the *P*1 asymmetric unit. The space group was determined as *C*2 after examining the symmetry of the calculated structure factors, so that there were 28 monomers in the *C*2 asymmetric unit.

General descriptions of the nodes generated by *phasertng.xtricorder* are given below.

#### 4.1.1. Crystal   

The DAG is rooted in the information about the molecules present in the crystallization drop: the sequence(s) of protein, DNA, RNA, ligands and small molecules from the crystallization conditions. A list of anomalously scattering elements (*e.g.* sulfur or selenium) is explicitly included. If known, the experimentally determined oligomeric association of the components in the unit cell will also be part of the hypothesis. Note that, at the root, the copy number of each component in the asymmetric unit is not part of the hypothesis and that the data analysis in *phasertng.xtricorder* is independent of the number of copies. In work to come, adding hypotheses about copy numbers will be a branch point on the DAG.

#### 4.1.2. Data   

More than one set of data may be obtained for a given set of crystal components, either for different crystal forms or for a single crystal form. Data collected from a single radiation-damaged crystal can be merged taking data up to different dosages, balancing damage against completeness and multiplicity. A multi-crystal data collection can result in data sets merged by including different subsets of crystals, balancing non-isomorphism against completeness and multiplicity. The processing of the diffraction data gives merged intensities, their errors, the unit-cell dimensions, the wavelength of data collection and the Laue symmetry.

#### 4.1.3. Anisotropy   

Systematic modulations of the intensities from an isotropic Wilson distribution caused by diffraction anisotropy are corrected by the application of anisotropic scaling factors. The anisotropic correction terms are unique for the point-group symmetry, with the anisotropy tensor constrained to that symmetry. If twinning is suspected and the data are expanded to lower symmetry (see below) the anisotropy correction need not be repeated in the lower symmetry space group with fewer constraints on the tensor, since the intensities will retain the higher symmetry.

#### 4.1.4. TNCS order   

Hypotheses about the TNCS can be ranked based on inspection of the Patterson map. The absence of TNCS is always considered, even when TNCS is indicated by the presence of large peaks in the Patterson map, as the results of our analysis indicate that large peaks in the Patterson map can be caused by crystal pathologies other than TNCS (Rye *et al.*, 2007 ▸; Dauter *et al.*, 2005 ▸).

#### 4.1.5. TNCS correction   

Systematic modulations of the intensities from an isotropic Wilson distribution caused by TNCS are accounted for by the application of expected intensity factors for each reflection derived from a model of the TNCS represented by the effective molecular radius, the r.m.s. deviation between TNCS-related components and the fraction of the scattering related by TNCS. The TNCS-correction terms depend on the hypothesis about the TNCS order.

#### 4.1.6. Twinning   

The probability of twinning is best determined after TNCS analysis, because accounting for the statistical effects of TNCS can unmask the effect of twinning on intensity statistics (Padilla & Yeates, 2003 ▸; Read *et al.*, 2013 ▸). If twinning is indicated, then the data may have been merged in a higher symmetry than the crystal symmetry, and subgroups of the space-group symmetry should be considered (as discussed below).

#### 4.1.7. Space group   

The space group is a hypothesis on a branch of the DAG. Ambiguities of space group within the Laue group arise from theoretical considerations (for example if the space group has subgroups and/or an enantiomorph) or on experimental grounds (for example if axial reflections were not recorded and hence systematic absences cannot be inspected). For SAD phasing in the case of an enantiomorphic space group, the enantiomorph of the space group is ambiguous when the anomalous substructure consists of a single type of anomalously scattering atom. For MR using single atoms as the model, the enantiomorph of the space group will not be resolved until the enantiomer of the structure can be interpreted. Perfect twinning can further complicate space-group determination by adding symmetry to the measured intensities.

#### 4.1.8. Space-group expansion   

Perfect twinning may mask the crystal symmetry by making the observed intensities consistent with a symmetry higher than that given by the crystal symmetry alone. The intensities can be merged in a Laue group higher than that of the Laue group of the crystal. The data may be expanded to the crystal symmetry without having to integrate the data using the crystal symmetry; there will be no loss of information if the data are perfectly twinned. After expanding the data, MR will give a set of solutions related by the twinning operator(s), which all model the intensities equally well.

### 4.2. Speed   

In order to compare the speed of *Phaser* and *Phasertng*, the *phasertng.xtricorder* algorithm was reduced to the functionality of *Phaser*’s NCS mode by removing the Padilla–Yeates *L*-test, propagating only the most probable TNCS order to the TNCS correction and not expanding the space group if twinning was detected.

The speed was compared for three different cases of TNCS (no TNCS, TNCS of order 2 and TNCS of order greater than 2) because each of these uses different TNCS-correction algorithms. With no TNCS, the only corrections to the intensities are anisotropic scaling terms. With TNCS of order greater than 2, in addition to the anisotropic scaling correction, TNCS-correction terms are derived from parameters for the TNCS order, the translation vector, the effective molecular radius, the r.m.s. deviation between TNCS-related components and the fraction of the scattering related by TNCS. With TNCS of order 2, in addition to these parameters, the orientational difference between the TNCS-related components is also a parameter in generating the TNCS-correction terms. The orientational difference is refined starting from exact alignment (no angular perturbation) and four small initial perturbations from perfect TNCS translation, giving five starting angles for refinement.

In *Phaser*’s NCS mode, there is no parallelization in the anisotropy correction or the outlier rejection and no parallelization for TNCS correction with TNCS of order greater than 2. For TNCS of order 2, *Phaser*’s NCS mode has coarse-grained parallelization of the TNCS correction over the five starting angles for refinement, leading to a maximum fivefold increase in speed even when more than five cores are available. Since the parallelization in *Phasertng* is over the reflections, *Phasertng* can utilize all available cores for parallel execution (assuming that the number of cores is fewer than the number of reflections).

Apart from the introduction of reflection-wise threading of anisotropy, outlier rejection and TNCS corrections, algorithmic changes also contributed to speed enhancements. Changes in the parameterization of the TNCS correction terms gave better convergence and also generally improved the results (Table 1 ▸). Changes to the minimization methods also improved the speed of convergence (Stockwell *et al.*, 2020 ▸) for the TNCS and anisotropy corrections. Because these details of the algorithms are not strictly the same, it is possible that when running without threading users may discover individual cases in which the runtime for *Phasertng* is slightly longer than that for *Phaser*.

#### 4.2.1. Database   

**Figure 3:**
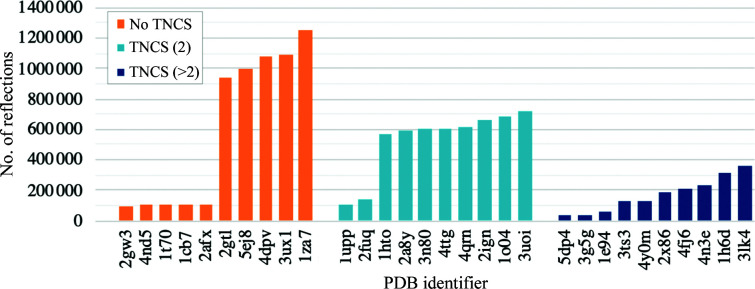
Number of reflections in each test case for the database of 30 test cases used for the speed tests in Figs. 4 ▸ and 5 ▸. PDB identifiers are shown for data with no TNCS (orange), for data with TNCS of order 2 (light blue) and data with TNCS of order greater than 2 (dark blue).

Speed tests were performed using a database of 30 test cases selected from the PDB where diffraction data had been deposited. All entries are found in the PDB-REDO database (Joosten *et al.*, 2012 ▸) and therefore the data can reproduce the published *R* factor within ten percentage points. Cases were selected following the curation of the data in Caballero *et al.* (2021 ▸). The entries in this database were sorted by number of reflections, and the selection was made by including some of those with the largest number of reflections in order to maximize the proportion of execution time spent in threaded sections of code (Fig. 3 ▸). Ten cases exercised the code for no TNCS (PDB entries 2gw3, 1cb7, 1t70, 4nd5, 2afx, 2gtl, 4dpv, 5ej8, 3ux1 and 1za7), ten exercised the code for TNCS of order 2 (PDB entries 2fuq, 1upp, 1hto, 2a8y, 4ttg, 2ign, 1o04, 3uio, 3n80 and 4qrn) and ten exercised the code for TNCS of order greater than 2 [PDB entries 5dp4 (4), 3ts3 (4), 3g5g (7), 1h6d (3), 4fj6 (6), 3lk4 (4), 2x86 (4), 1e94 (6), 4y0m (4) and 4n3e (7), where the TNCS order is given in parentheses].

#### 4.2.2. Hardware and OS   

Calculations were performed on a multiprocessing workstation with two eight-core hyperthreaded Intel Xeon processors W-2145 at 3.70 GHz and 128 GB RAM with operating system Centos 7. Compilation was with GCC 4.8.5 (C++11 flag) with optimization level 3. Differences between runtimes for *Phaser* executables compiled on Linux and Windows operating systems, with and without patches for the ‘meltdown’ bug, have recently been explored (Oeffner, 2018 ▸) and we would expect these conclusions to also hold for *Phasertng*.

### 4.3. Results   

**Figure 4:**
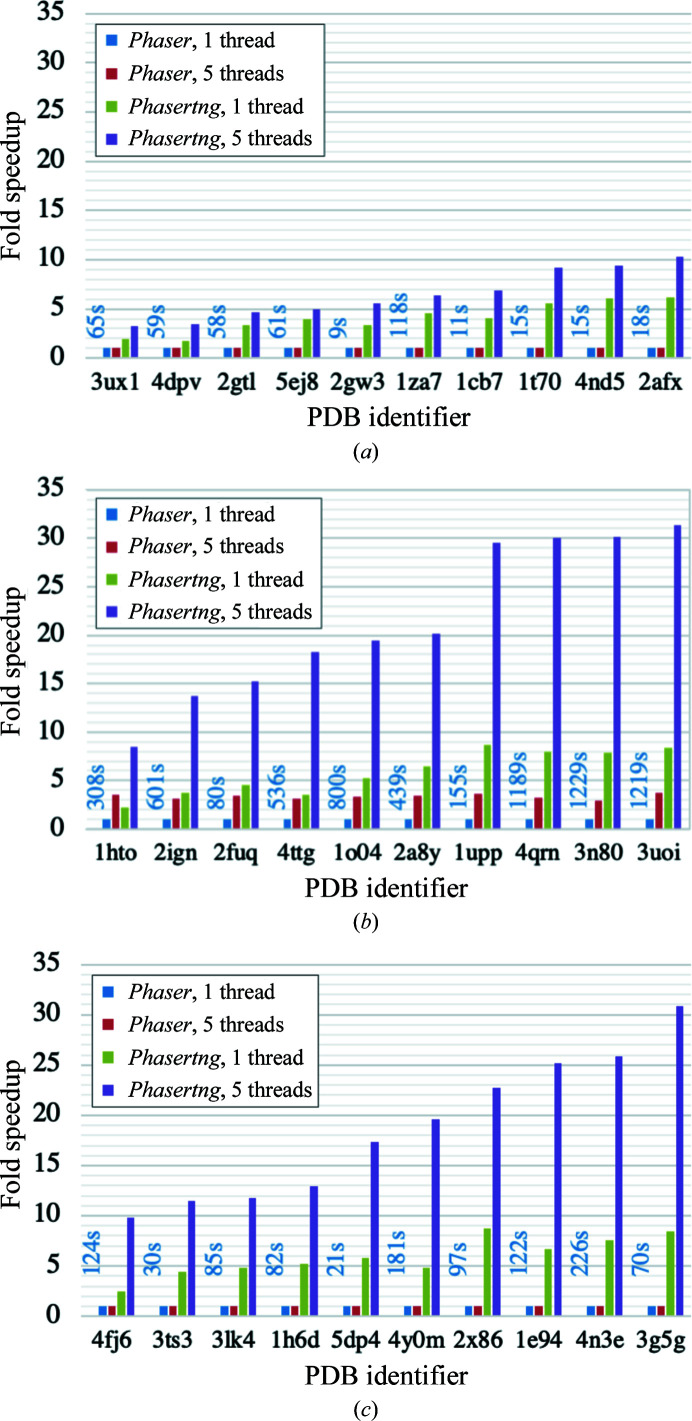
Comparison of the fold speedup of wall time for *Phaser* and *Phasertng* with and without threading over five threads. (*a*) Data with no TNCS, (*b*) data with TNCS of order 2 and (*c*) data with TNCS of order greater than 2. Four times are shown for each PDB identifier: *Phaser* without threading (blue), *Phaser* threaded on five cores (red), *Phasertng* without threading (green) and *Phasertng* threaded on five cores (purple). The longest runtime for each PDB identifier is shown in seconds above each column group, which is for *Phaser* without threading in every case.

**Figure 5:**
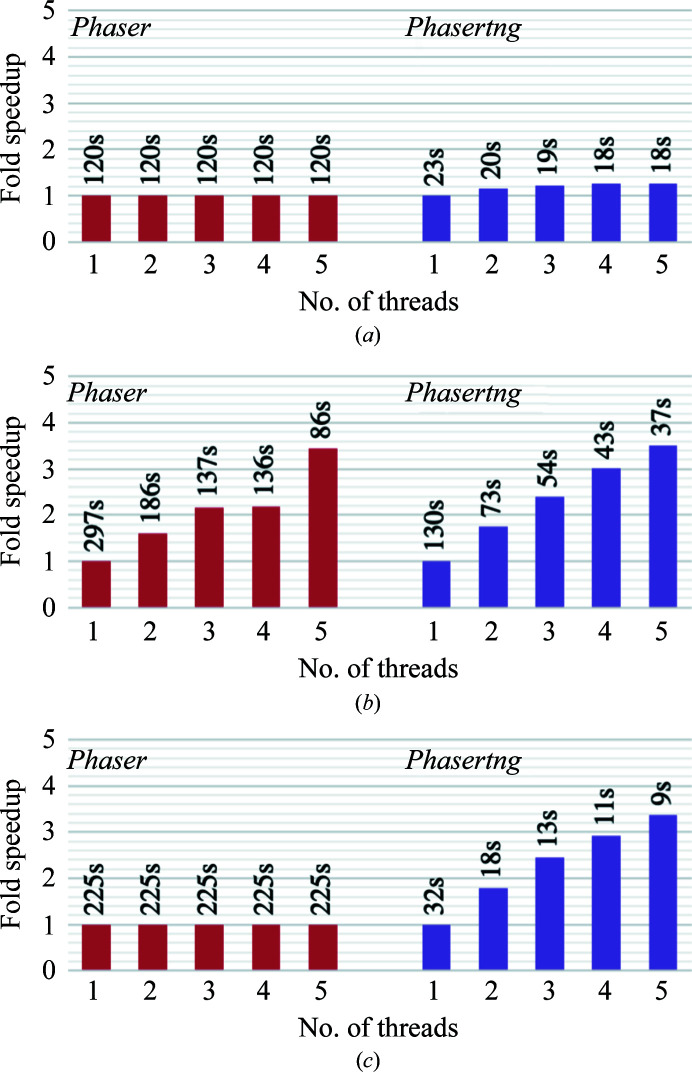
Comparison of the fold speedup of wall time for *Phaser* (red) and *Phasertng* (purple) with threading for between one and five threads. The elapsed wall time is shown above each column in seconds for (*a*) data with no TNCS (PDB entry 1za7), (*b*) data with TNCS of order 2 (PDB entry 1hto) and (*c*) data with TNCS of order greater than 2 (PDB entry 4n3e).

The improved algorithms, memory management and C++11 threading implemented over reflection loops discussed above resulted in significant speed enhancements in *Phasertng* over *Phaser* (Fig. 4 ▸). Calculations were performed using one and five threads, where five was chosen because of the upper limit on the increase in speed possible with the *Phaser* threading (see above). Average speed improvements are shown in Table 2 ▸. The *Phasertng* code without threading runs between threefold and eightfold faster than the broadly equivalent *Phaser* code. With threading, the total runtime (which includes the execution of small sections of non-threaded code) runs between fivefold and 30-fold faster when using five threads. The dependence of runtime on the number of threads is shown for one case each of no TNCS, TNCS of order 2 and TNCS of order greater than 2 (Fig. 5 ▸). The fold speedup is proportional to the number of threads for up to five threads (Fig. 5 ▸). In *Phaser*, the fold speedup cannot increase with more than five threads owing to the coarse-grained parallelization discussed previously. In *Phasertng*, the fold speedup can increase with more than five threads, although the fold speedup does not continue to increase almost linearly with thread number owing to thread-initialization overheads and nonthreaded code running in serial mode. In future work, the optimal number of threads of *phasertng.xtricorder* will be set by embedding it in a control structure that can take account of the number of reflections and the overall load balance on their system, after benchmarking.

## 5. Discussion   

*Phasertng* has supplanted *Phaser* as our platform for implementing novel phasing algorithms and bringing the most effective approaches to the crystallographic community. The change between *Phaser* and *Phasertng* can be summarized as pivoting the focus of the software from algorithms that generate results in *ad hoc* data structures, which must then be interpreted by automation pipelines, to an extensible graph database structure describing automation, whose nodes are filled with data by the software. Our goal remains achieving the best possible initial electron-density map for model building by MR and SAD phasing.

Data tracking and job management are major components of the two software distributions through which *Phaser* is distributed: *CCP*4 (Winn *et al.*, 2011 ▸) and *Phenix* (Liebschner *et al.*, 2019 ▸). In *CCP*4, *Phaser* has been integrated into the data-tracking systems in *ccp*4*i* (no longer supported; Potterton *et al.*, 2003 ▸), *ccp*4*i*2 (Potterton *et al.*, 2018 ▸) and *CCP*4 Cloud (Krissinel *et al.*, 2018 ▸), while in *Phenix* there are several *Phaser* interfaces (Echols *et al.*, 2012 ▸). Support for directed acyclic graphs in *Phasertng* will supplement these data-tracking and job-management systems by giving them a new tool with which to report complicated *Phasertng*-dependent phasing strategies. In particular, directed acyclic graphs have mathematical properties that allow the execution of useful algorithms over their nodes, for example topological sorting, the ability to compute a path between any pair of nodes and fast algorithms for calculating the shortest path (Cormen *et al.*, 1990 ▸).

We hope that shifting to the *Phasertng* codebase will also benefit software that is currently dependent on *Phaser*. In the *Phenix* suite there are *phenix.automr* (Zwart *et al.*, 2008 ▸) and *phenix.mr_rosetta* (Terwilliger *et al.*, 2012 ▸); in the *CCP*4 suite (Winn *et al.*, 2011 ▸) there are *MrBUMP* (Keegan & Winn, 2007 ▸, 2008 ▸), *SIMBAD* (Simpkin *et al.*, 2018 ▸), *AMPLE* (Bibby *et al.*, 2012 ▸; Thomas *et al.*, 2015 ▸) and *MRparse* (https://github.com/rigdenlab/MrParse); in the *ARCIMBOLDO* suite there are *ARCIMBOLDO*, *ARCIMBOLDO_LITE*, *ARCIMBOLDO_BORGES* and *ARCIMBOLDO_SHREDDER* (Rodríguez *et al.*, 2009 ▸; Sammito *et al.*, 2013 ▸, 2014 ▸, 2015 ▸; Millán *et al.*, 2018 ▸). *Auto-Rickshaw* (Panjikar *et al.*, 2005 ▸) automates structure solution by experimental phasing and MR using *Phaser*. *Phaser* is also used as the engine behind the *UCLA Diffraction Anisotropy Server* (Strong *et al.*, 2006 ▸) and the *SBGrid* wide-search MR server (Stokes-Rees & Sliz, 2010 ▸). Diamond Light Source has the *Phaser*-dependent difference-map pipeline *DIMPLE* for ligand screening (Wojdyr, 2018 ▸). We expect that there are bespoke pipelines using *Phaser* for specific purposes in laboratory and synchrotron settings of which we are not aware.

A simplistic approach to phasing is best described as a disjoint union of DAGs: every MR or SAD phasing trial is treated independently. More sophisticated search strategies simultaneously consider results from searches with different MR models or SAD substructures or both, as in MR-SAD. The *phenix.MRage* pipeline processes many MR models in parallel and if a solution is found with one model then all models are superimposed on the solution and rescored, so that the best model can be used to phase the map put forward for model building (Bunkóczi *et al.*, 2013 ▸). The *ARCIMBOLBO* software makes high-level use of persistence of solutions while the model is systematically varied (Rodríguez *et al.*, 2009 ▸; Sammito *et al.*, 2013 ▸, 2014 ▸, 2015 ▸; Millán *et al.*, 2018 ▸). The molecular-replacement parameter matrix (MRPM) procedure uses the anomalous substructure derived from MR-SAD to verify MR substructures (Pedersen *et al.*, 2016 ▸). Other examples of complicated phasing strategies include *ab initio* phasing using molecular averaging (Tsao *et al.*, 1992 ▸), phase improvement by cross-crystal averaging after MR (Isupov *et al.*, 2004 ▸) or experimental phasing (Crennell *et al.*, 2000 ▸; Chen *et al.*, 2005 ▸; Su *et al.*, 2010 ▸), and phasing with electron-microscopy reconstructions (Wynne *et al.*, 1999 ▸). These approaches will be simplified with native support for the DAG data structure in *Phasertng*.

*Phasertng* is central to the development of *phaser.voyager*, which will leverage the solution-tracking capabilities of the DAG in sophisticated and exhaustive phasing pathways. The DAG, paired with a formal database architecture for efficient model storage and retrieval, will streamline the termination and restarting of phasing pathways in order to simplify user intervention in search strategies. Wrapping *Phasertng* and the DAG data structure in *phaser.voyager* enables us to make optimal use of the maximum-likelihood and multivariate statistics for the preparation and selection of the data, the choice of SAD or MR as the primary phasing strategy, the generation of ensemble models customized to both the data and the hypothesis of the contents of the unit cell, tracking the persistence of solutions, managing coordinate editing and refinement. By applying graph analysis and data-mining methods to databases of *phaser.voyager*-generated DAGs, we aim to improve the efficiency of phasing and discover unexpected dependencies between DAG node data elements. Details of the *phaser.voyager* pipeline will be published elsewhere.

*Phasertng*, *phasertng.xtricorder* and *phaser.voyager* will be made available through the *Phenix* (Liebschner *et al.*, 2019 ▸) and *CCP*4 (Winn *et al.*, 2011 ▸) software distributions.

## Figures and Tables

**Table 1:** Comparison of the translational noncrystallographic symmetry (TNCS)-correction algorithms in *Phaser* and *Phasertng* Second moments of the intensity distributions and *P*-value for twinning after TNCS expected intensity-factor correction terms have been applied for the ten cases of TNCS of order 2 and of TNCS of order greater than 2. The expected values of the second moments for untwinned acentric and centric data are 2.0 and 3.0, respectively; the corresponding values for perfectly twinned data are 1.5 and 2.0, respectively.

		*Phaser*				*Phasertng*			
		Second moments		*P*-value		Second moments		*P*-value	
PDB code	TNCS order	Centric	Acentric	Untwinned	Twin α < 5%	Centric	Acentric	Untwinned	Twin α < 5%
1hto	2	3.09	2.03 ± 0.008	1	1	3.05	2.03 ± 0.006	1	1
1o04	2	2.94	1.98 ± 0.008	0.00266	1	2.95	1.95 ± 0.006	2.51 × 10^−20^	1
1upp	2	2.52	1.72 ± 0.021	6.16 × 10^−40^	1.41 × 10^−10^	2.62	1.70 ± 0.014	1.26 × 10^−102^	6.89 × 10^−49^
2a8y	2	—	2.03 ± 0.010	1	1	—	2.02 ± 0.006	1	1
2fuq	2	3.03	2.00 ± 0.024	1	1	2.97	1.98 ± 0.013	0.0516	1
2ign	2	3.02	1.99 ± 0.009	0.178	1	2.87	1.87 ± 0.006	2.15 × 10^−117^	1.29 × 10^−9^
3n80	2	3.03	2.03 ± 0.009	1	1	3.02	2.03 ± 0.009	1	1
3uio	2	—	1.65 ± 0.007	0	3.44 × 10^−272^	—	1.65 ± 0.007	0	3.58 × 10^−278^
4qrn	2	3.22	2.07 ± 0.011	1	1	3.22	2.07 ± 0.011	1	1
4ttg	2	2.96	2.03 ± 0.009	1	1	2.99	2.04 ± 0.006	1	1
1e94	6	3.71	2.31 ± 0.028	1	1	3.75	2.33 ± 0.028	1	1
1h6d	3	3.53	2.33 ± 0.017	1	1	3.58	2.28 ± 0.008	1	1
2x86	4	3.48	2.51 ± 0.015	1	1	3.47	2.46 ± 0.010	1	1
3g5g	7	2.75	2.06 ± 0.034	1	1	2.58	2.01 ± 0.025	1	1
3lk4	3	—	2.35 ± 0.014	1	1	—	2.43 ± 0.007	1	1
3ts3	4	3.65	2.19 ± 0.020	1	1	3.62	2.12 ± 0.013	1	1
4fj6	6	—	2.01 ± 0.019	1	1	—	1.95 ± 0.010	1.95 × 10^−7^	1
4n3e	7	7.13	3.21 ± 0.014	1	1	4.44	2.47 ± 0.009	1	1
4y0m	6	3.20	2.05 ± 0.017	1	1	3.19	2.04 ± 0.017	1	1
5dp4	4	4.18	2.67 ± 0.056	1	1	4.29	2.63 ± 0.056	1	1

**Table 2:** Comparison of the average fold speedup of *Phasertng* over *Phaser* for the test cases shown in Fig. 3 ▸

	Without threading, one core	With threading, five cores
No TNCS	4.1 ± 1.6	6.4 ± 2.5
TNCS of order 2	5.1 ± 2.3	21.6 ± 8.1
TNCS of order greater than 2	5.9 ± 2.0	18.8 ± 7.2
